# Der Europäische MSM Internet Survey als Grundlage für die Präventionsarbeit in Deutschland für Männer, die Sex mit Männern haben

**DOI:** 10.1007/s00103-021-03429-3

**Published:** 2021-10-13

**Authors:** Ulrich Marcus, Susanne B. Schink

**Affiliations:** grid.13652.33 0000 0001 0940 3744 Abt. Infektionsepidemiologie, Robert Koch-Institut, Seestraße 10, 13353 Berlin, Deutschland

**Keywords:** Syndemie, Sexuelle und psychische Gesundheitsprobleme, Substanzkonsum, HIV, Sexuell übertragbare Infektionen, Syndemic, Sexual and mental health problems, Substance use, HIV, Sexually transmitted infections

## Abstract

**Hintergrund:**

Sexuelle Minderheiten sind in hohem Maße anfällig für sexuell übertragbare Infektionen (STI), psychische Probleme wie Depressionen und Suizidalität sowie für gesundheitliche und soziale Probleme, die mit Substanzkonsum assoziiert sind.

**Ziel der Arbeit:**

Wir beschreiben ausgewählte Ergebnisse aus dem Europäischen MSM Internet Survey (EMIS) 2017, eine der größten Onlinebefragungen von Männern, die Sex mit Männern haben (MSM), und diskutieren deren Implikationen für die Prävention in Deutschland.

**Material und Methoden:**

Das übergeordnete Ziel von EMIS-2017 war es, Daten für die Planung von HIV- und STI-Präventions- und -Behandlungsprogrammen zu erheben. Die Zielpopulation waren in Europa lebende MSM. Der Fragebogen enthielt Fragen zu Demografie, Morbiditäten, Verhalten, Bedarfen und Interventionen. Die deutschen Teilnehmer wurden von 10/2017 bis 01/2018 vor allem über 2 große Onlinedatingplattformen rekrutiert.

**Ergebnisse und Diskussion:**

EMIS-2017 zeigt, dass MSM stark von psychischen Belastungen und STI betroffen sind. Jüngere MSM leiden verstärkt unter depressiven Störungen und Suizidalität, während bei älteren MSM der Konsum von Alkohol und anderen psychoaktiven Substanzen bedeutsam ist.

MSM sind von STI stärker betroffen als Heterosexuelle. Viele STI verursachen keine oder nur untypische Beschwerden, daher wird ein Screening auf der Basis einer Risikoeinschätzung favorisiert. Für einen Teil der STI gibt es bislang keine medizinischen und/oder Public-Health-Erkenntnisse, die für eine Behandlung asymptomatischer Infektionen sprechen. EMIS-2017 identifizierte Bedarfe nach Untergruppen in Bezug auf Wissenslücken zu HIV-Post- und HIV-Präexpositionsprophylaxe (PEP, PrEP), Erreichbarkeit durch Informations- und Präventionsangebote sowie unzureichende Nutzung dieser Angebote.

**Zusatzmaterial online:**

Zusätzliche Informationen sind in der Online-Version dieses Artikels (10.1007/s00103-021-03429-3) enthalten.

## Abstract (en)

**Background:**

Sexual minorities are highly vulnerable to sexually transmitted infections (STIs), mental health problems such as depression and suicidality, and substance use-associated health and social problems.

**Research question:**

We describe selected findings from the European MSM Internet Survey (EMIS) 2017, one of the largest online surveys of men who have sex with men, and discuss their implications for prevention in MSM in Germany.

**Materials and methods:**

The overall aim of EMIS-2017 was to collect data for planning HIV and STI prevention and treatment programs. The target population were men living in Europe who have sex with men and/or are attracted to men. The questionnaire contained questions on demography, morbidities, behaviour, needs and interventions. Participants were recruited mainly via two popular online dating platforms from 10/2017 to 01/2018.

**Results and discussion:**

EMIS-2017 shows that MSM are severely affected by psychological stress and STIs. In terms of psychological stress, younger MSM suffer in particular from depressive disorders and suicidality, while the use of alcohol and other psychoactive substances is of concern among older MSM.

MSM are more often affected by STIs than the heterosexual majority population. Many STIs are a‑ or pauci-symptomatic; thus, screening based on risk-assessment is favoured to detect infections. However, for the time being, for some STIs there is no medical and/or public health evidence to support treatment of asymptomatic infections. EMIS-2017 identified needs by subgroups as far as knowledge gaps on PEP (HIV post-exposure prophylaxis) and PrEP (HIV pre-exposure prophylaxis), being reached by information and prevention offers as well as insufficient uptake of such offers.

## Hintergrund

Sexuelle Minderheiten, die aufgrund ihrer sexuellen Orientierung und Selbstidentifikation von der heterosexuellen Norm abweichen, waren und sind in den meisten Gesellschaften weltweit sozial und oft auch rechtlich diskriminiert. Dies resultiert in Minderheitenstress [1–3], erschwert die Partnersuche und den Aufbau stabiler, sozial akzeptierter Partnerschaften, begünstigt flüchtige und häufig wechselnde Partnerschaften und hohe Partnerzahlen und wirkt sich zudem negativ auf die psychische Gesundheit, das Selbstwertgefühl und den Zugang zu einer auf die Bedürfnisse zugeschnittenen Gesundheitsversorgung aus. Gewalt- und Missbrauchserfahrungen sowie kompensatorischer Substanzgebrauch sind häufig. Im Sinne einer *Syndemie, *also einer Epidemie, die durch gleichzeitiges Auftreten von Krankheiten und sozialen Problemen geprägt ist, tragen diese Faktoren zu einer z. T. deutlich erhöhten Vulnerabilität gegenüber sexuell übertragbaren Infektionen (STI), psychischen Gesundheitsproblemen wie Depressionen und Suizidalität und substanzkonsumassoziierten gesundheitlichen und sozialen Problemen bei [4–13].

Die Erforschung und Prävention dieser Belastungen wird dadurch erschwert, dass die Zugehörigkeit betroffener Personen zu bestimmten marginalisierten Gruppen in Einrichtungen der Gesundheitsversorgung oft schlecht erkennbar ist, nicht zuletzt aufgrund negativer Diskriminierungserfahrungen im Gesundheitssystem [2, 14, 15].

Ein häufig eingesetztes Instrument zur Erhebung von Präventionsbedarfen sind sog. KABP-Surveys („knowledge, attitudes, behaviours and practices“; [16]). Die Rekrutierung von Teilnehmern erfolgte häufig über klinische Versorgungseinrichtungen, an szeneaffinen Treffpunkten (Bars, Klubs, Saunen etc.) oder über Schneeballverfahren, die soziale Netzwerke innerhalb der Gruppen nutzen. Mit der Verbreitung des Internets und sozialer Medien wurden diese durch Onlinerekrutierung ergänzt bzw. ersetzt.

Zu beachten ist bei allen genannten Rekrutierungsmethoden, dass sie in der Regel nichtrepräsentative Stichproben ergeben, die aufgrund ihres Teilnahmebias ein mehr oder weniger stark verzerrtes Bild der untersuchten Population vermitteln [17–19]. Herkömmliche populationsbezogene Stichproben, die auf Repräsentativität für die Bevölkerung ausgelegt sind, eignen sich nur sehr begrenzt für detailliertere Untersuchungen von Minderheiten, da sie in den Stichproben meist nur in kleiner Zahl vertreten sind, ihre eindeutige Identifizierung oft nicht möglich ist und spezifische Fragestellungen für diese Gruppen nur schwer in die Studiendesigns integriert werden können.

Vor diesem Hintergrund beschreiben wir hier die Konzeption und ausgewählte Ergebnisse einer der größten Onlinebefragungen von Männern, die Sex mit Männern haben (MSM), des Europäischen MSM Internet Survey (EMIS) 2017 [20, 21]. Große, niedrigschwellig angelegte Onlinebefragungen wie EMIS-2017 haben den Vorteil, dass sie bei ausreichend hohen Teilnehmerzahlen auch Aussagen zu Teilgruppen (Minderheiten innerhalb der Minderheit) gestatten und aufgrund der Vielsprachigkeit des Erhebungsinstruments und der internationalen Rekrutierungsstrategie internationale Vergleiche und die Erkennung strukturell und policy-bedingter Unterschiede zwischen Ländern erlauben. Wir fokussieren hier auf die in Deutschland rekrutierte Stichprobe und diskutieren die Nutzbarkeit der Ergebnisse für die Präventionsarbeit für MSM in Deutschland.

## Methoden

### Zielpopulation und Ziele der Befragung

EMIS-2017 war ein Teil von ESTICOM (Europäische Umfragen und Schulungen zur Verbesserung der Gesundheit der MSM-Community), einem dreijährigen Projekt (2016–2019), das vom Gesundheitsprogramm der Europäischen Kommission 2014–2020 finanziert wurde [22]. In der Ausschreibung wurde eine Bestandsaufnahme der sexuellen Gesundheit von schwulen, bisexuellen und anderen Männern, die Sex mit Männern haben, in Europa gefordert.

Das übergeordnete Ziel von EMIS-2017 bestand darin, Daten zu generieren, die für die Planung von HIV- und STI-Präventions- und -Behandlungsprogrammen und die Überwachung der nationalen Fortschritte in diesem Bereich nützlich sind. Dazu wurden das Ausmaß und die Verteilung des HIV-Übertragungsrisikos und des Schutzverhaltens sowie die damit verbundenen HIV-Präventionsbedürfnisse beschrieben, auch unter Berücksichtigung des selbstberichteten STI-Testverhaltens, der Testdurchführung und verschiedener STI-Diagnosen, einschließlich Virushepatitis.

Die Zielpopulation und Ein- bzw. Ausschlusskriterien von EMIS-2017 waren Männer:

- die in Europa leben,
- im oder über dem Alter der Einwilligungsfähigkeit in homosexuelle Handlungen in dem Land, in dem sie leben,
- die sich sexuell zu Männern hingezogen fühlen und/oder Sex mit Männern hatten und
- die angaben, dass sie Art und Zweck der Studie verstehen und der Teilnahme zustimmen.

Auf Länderebene sollte die MSM-Umfrage Daten zum Verständnis der Bedürfnisse der Population und zur Steuerung von Präventionsprogrammen generieren. International können Muster politischer und kultureller Gegebenheiten sowie der Angebotsstrukturen auf die Ausbreitung und Eindämmung von Epidemien untersucht werden. So können auch Erkenntnisse gewonnen werden, die mit rein nationalen oder lokalen Studien nicht möglich sind [21].

### Fragebogendesign

Der Fragebogen basiert auf einer früheren Version der Umfrage (EMIS-2010; [23]). Für die Überarbeitung erfolgte ein Scoping-Review der seit EMIS-2010 veröffentlichten verfügbaren MSM-Umfragen, gefolgt von 3 Konsultationen aller europäischen Partner, einem Vortest und der Pilotierung der Onlineversion in englischer Sprache.

### Inhalt des Fragebogens

Die endgültige Fragebogenversion enthielt die folgenden Themenblöcke (s. auch Onlinematerial zu diesem Artikel):

#### Demografie.

34 beschreibende Elemente. Unter anderem Angaben zu Geschlechtsidentität, Alter, Wohnsitz- und Geburtsland, Ortsgröße des Wohnorts, Dauer der Ausbildung, Partnerschaftsstatus sowie zur gefühlten Einkommenssituation und Zugehörigkeit zu einer ethnischen Minderheit.

#### Morbiditäten.

16 Fragen zum Gesundheitszustand. Dazu gehören 4 Fragen zu psychischer Gesundheit (einschließlich Angst/Depression (Fragebogen zur Patientengesundheit Patient Health Questionnaire PHQ-4) [24], Selbstmordgedanken, sexueller (Un)Zufriedenheit, Alkoholabhängigkeit (CAGE-4) [25]) sowie 12 Fragen zu selbstberichteten Diagnosen von Infektionen mit HIV, Syphilis, Gonorrhö, Chlamydien, anogenitalen Warzen sowie Hepatitis A, B und C.

#### Verhalten.

83 Fragen zu Handlungen, die Risiken bergen oder Vorsichtsmaßnahmen beeinträchtigen. Dazu gehören 42 Fragen zum sexuellen Verhalten (einschließlich des ersten und letzten Geschlechtsverkehrs mit Männern; feste und nicht feste männliche Sexualpartner im letzten Jahr; Kondomgebrauch; Sex mit Frauen; letzter Sex mit einem nicht festen männlichen Partner), 20 Fragen zum Substanzkonsum (einschließlich Alkohol- und Tabakprodukte und illegale Substanzen) und 5 zum injizierenden Drogenkonsum, 6 zur Kombination von Sex und Drogen (Chemsex), jeweils 2 zum Bedarf und der Verwendung von HIV-Postexpositionsprophylaxe (PEP) und HIV-Präexpositionsprophylaxe (PrEP), 4 zu HIV-Tests und -Behandlungen, 2 zum Immunstatus von Hepatitis A und B und 2 zur Benachrichtigung von Sexpartnern nach einer Syphilis- oder Gonorrhödiagnose.

#### Bedarfe.

22 Fragen zu Möglichkeiten, Befähigungen und Motivationen für Risiko- und Schutzverhalten. Dazu gehörten 2 validierte Skalen zu Verhaltensweisen (Skala für soziale Unterstützung [SPS] und internalisierte Homonegativität; [26, 27]), 4 Aussagen zur Ermittlung des Wissens zu Safer Sex, jeweils 3 zu PEP und PrEP und 5 zu HIV-Tests und -Behandlungen, eine Reihe von Bedarfen in Bezug auf Substanzkonsum, 3 Aussagen zu Virushepatitis und eine über STI.

#### Interventionen.

35 Fragen zu den Handlungen anderer, die Bedürfnisse erfüllen oder untergraben. 3 Fragen zu homophobem Missbrauch (siehe Onlinematerial Abschn. 4.5), 2 über den Zugang zu Kondomen, 3 zum Zugang zur Drogenberatung, 6 zu PrEP-bezogenen Angeboten, eine zu HIV-/STI-Informationen, 7 zu HIV- und 12 zu STI-Testangeboten sowie eine zu dem Impfangebot gegen Virushepatitiden.

### Teilnehmerrekrutierung

Die Umfrage war in 33 Sprachen verfügbar. Die Sprache konnte frei gewählt werden [21]. Zum Befragungszeitpunkt 2017 waren Smartphone-Apps ein verbreitetes Medium zur Kontaktaufnahme unter MSM. Der Fragebogen konnte direkt am Smartphone ausgefüllt werden. Die Teilnehmerwerbung für EMIS-2017 erfolgte auf:

- Websites unterstützender Organisationen,
- allgemeinen sozialen Netzwerken wie Facebook, Twitter und Instagram,
- Dating-Apps auf Smartphones und Webseiten für MSM.

Die Onlinerekrutierung von Teilnehmern begann am 18.10.2017 und endete am 31.01.2018.

## Ergebnisse

### Stichprobenbeschreibung

An EMIS-2017 beteiligten sich 23.107 Männer, die zum Befragungszeitpunkt in Deutschland lebten. Männlich zu sein, war eine Voraussetzung für die Teilnahme. Insgesamt 99 % definierten sich als „männlich“ und 1 % betrachteten sich als „Transmänner“. Transmänner, die Sex mit Männern angegeben hatten, konnten teilnehmen, Transfrauen wurden aus dem Untersuchungssample ausgeschlossen. Das Durchschnittsalter (Median) betrug 36 Jahre (Bereich 14 bis 100, Mittelwert 37,2, Standardabweichung 12,8). Das Durchschnittsalter variierte je nach Bundesland, vermutlich primär infolge von Binnenmigration. 13 % der zum Befragungszeitpunkt in Deutschland lebenden Teilnehmer wurden nicht in Deutschland geboren. Eine Ausbildung jenseits von 16 Jahren hatten 97 % der Befragten durchlaufen (mittlere Anzahl 6 Jahre) und 89 % befanden sich 2 oder mehr Jahre nach dem 16. Lebensjahr in Ausbildung. Erwerbstätig waren 72 % und diese mehrheitlich vollzeitbeschäftigt. Mehr als jeder 20. war arbeitslos und es gab einen beträchtlichen Anteil von Studierenden (14 %). Finanzielle Probleme wurden von 17 % angegeben, 34 % bezeichneten ihre finanzielle Situation als ausreichend und 49 % gaben an, dass ihre finanzielle Situation gut oder sehr gut war.

#### Sexuelle Anziehung, Selbstdefinition, Outness und Partnerschaften.

Von niemandem sexuell angezogen fühlten sich weniger als 1 %; 5 % fühlten sich von nichtbinären Personen (eine Kategorie für Geschlechtsidentitäten, die nicht ausschließlich männlich oder weiblich sind) angezogen, 16 % von Frauen und 99 % von Männern. Alle, die nicht angaben, von Männern angezogen zu sein, hatten zuvor Sex mit Männern gehabt. Als schwul oder homosexuell bezeichneten sich 77 %, als bisexuell 16 %. 59 % der Männer berichteten, dass die Mehrheit der Menschen, die sie kannten, über ihre sexuelle Orientierung Bescheid wusste. Eine/n festen Partner/feste Partnerin hatten zum Befragungszeitpunkt 39 %, am häufigsten einen männlichen Partner (31 % aller Teilnehmer). 54 % waren zum Befragungszeitpunkt Single. Für Sex bezahlt hatten mehr Teilnehmer als Geld für Sex genommen, sowohl in ihrem ganzen bisherigen Leben (18 % gegenüber 15 %) als auch in den letzten 12 Monaten (10 % gegenüber 5 %). Die Mehrheit, die in den letzten 12 Monaten Sex verkauft oder gekauft hatte, hatte dies nur ein- oder zweimal getan.

### Morbiditäten

Morbidität beschreibt die Betroffenheit von physischen und psychischen Erkrankungen. Für die gesundheitsbezogene Präventionsarbeit sind die Verringerung und Vorbeugung von Erkrankungen die ultimativen Ziele. EMIS-2017 fragte zu psychischen Erkrankungen und STI.

#### Angst und Depression.

Unter Verwendung des PHQ‑4 berichteten 13 % über mindestens mäßige Angstzustände bzw. Depressionen in den letzten 2 Wochen und 5 % über schwere Angstzustände und Depressionen.

#### Selbstmordgedanken.

16 % hatten in den letzten 2 Wochen daran gedacht, sich selbst zu verletzen, und 4 % an mindestens der Hälfte der Tage in dieser Zeit.

#### Sexuelle Unzufriedenheit.

23 % gaben auf einer 10-Punkte-Selbstbewertungsskala fehlende sexuelle Zufriedenheit an.

#### Alkoholabhängigkeit.

Gemäß des CAGE-4-Screening-Instruments erfüllten 22 % die Kriterien für eine mögliche Alkoholabhängigkeit (CAGE = Akronym für **C**utting down, **A**nnoyed, feeling **g**uilty, need for **e**ye-opener).

#### HIV-Diagnosen.

11 % der gesamten Stichprobe gaben an, jemals mit HIV diagnostiziert worden zu sein. Ohne die Teilnehmer, die bereits länger als 12 Monate diagnostiziert waren, betrug der Anteil derjenigen, die in den letzten 12 Monaten eine HIV-Erstdiagnose erhalten hatten, 1,1 %.

#### Diagnostizierte HIV-Infektion mit nachweisbarer Viruslast.

1 % der Stichprobe berichteten von einer HIV-Infektion mit nachweisbarer Viruslast.

#### Diagnose von STI.

3 % wurden in den letzten 12 Monaten mit Syphilis diagnostiziert, 4 % mit Gonorrhö und 4 % mit Chlamydien oder Lymphogranuloma venereum (LGV).

#### Diagnose von Anal- oder Genitalwarzen.

(Infektion mit dem humanen Papillomavirus, HPV) 16 % berichteten jemals Anal‑/Genitalwarzen.

#### Hepatitis.

7 % berichteten eine Infektion mit Hepatitis A, 5 % Hepatitis B und 2 % Hepatitis C. Etwas mehr als 1 % der Stichprobe berichteten von einer Koinfektion mit HIV und Hepatitis.

### Risiko- und Schutzverhalten

Unter Risiko- und Schutzverhalten subsumieren wir Verhalten, das zu den oben beschriebenen Erkrankungen beiträgt bzw. vor diesen schützt. Wir fragten nach 2 Aspekten von Risikoverhalten (Sex haben, Drogen nehmen und die Kombination aus beidem) und 4 Verhaltensweisen, die vor Erkrankungen schützen (antiretrovirale Medikamente nehmen, Informationen zum HIV-Status austauschen, Kondome benutzen und Impfungen in Anspruch nehmen).

#### HIV-Diagnose und Behandlung.

11 % gaben an, jemals mit HIV diagnostiziert worden zu sein. 95 % dieser Personen nahmen zum Befragungszeitpunkt antiretrovirale Therapien (ART) ein.

#### PEP.

Unter den Teilnehmern, bei denen kein HIV diagnostiziert war, hatten 5 % jemals versucht eine PEP zu erhalten, 3 % hatten jemals eine PEP eingenommen. Dies bedeutet, dass 32 % derjenigen, die eine PEP erhalten wollten, diese nicht bekamen.

#### PrEP.

Unter den Teilnehmern, bei denen kein HIV diagnostiziert war, verwendeten 2 % zum Befragungszeitpunkt PrEP und 2 Drittel von ihnen täglich.

#### Hepatitis-A- und -B-Impfung.

38 % waren gemäß ihrer Selbstangaben potenziell nicht geschützt vor Hepatitis-A-Infektionen und 39 % nicht vor Hepatitis-B-Infektionen.

#### Sex mit Männern.

In den letzten 12 Monaten berichteten 81 % Analverkehr und davon 61 % kondomlosen Analverkehr mit einem Mann.^1^

#### Sex mit Frauen.

48 % hatten jemals Sex mit einer Frau, 19 % in den letzten 5 Jahren und 12 % im letzten Jahr. Der Gebrauch von Kondomen beim Geschlechtsverkehr mit Frauen war polarisiert: 43 % benutzten sie nie und 31 % benutzten sie immer.

#### Alkohol und Tabak.

Alkohol war in allen Zeitintervallen die am häufigsten konsumierte Substanz, mit nahezu universellem Lebenszeitkonsum (97 %) und 73 % in der Vorwoche. Insgesamt 34 % hatten in den letzten 24 h Tabak konsumiert und 39 % in der Vorwoche.

#### Konsum illegaler Substanzen.

Die am häufigsten konsumierte nicht legale Substanz war Cannabis, das von 44 % jemals und in den letzten 4 Wochen von 12 % konsumiert wurde. 4 weitere Substanzen wurden von 10–20 % der Befragten jemals eingenommen: Kokain, Ecstasy, Amphetamin und Gamma-Hydroxybutyrat/Gamma-Butyrolacton (GHB/GBL). Von anderen Substanzen (Ketamin, LSD, Crystal Meth, Mephedron, synthetische Cannabinoide, andere synthetische Stimulanzien, Heroin und Crack-Kokain) war in den letzten 4 Wochen keine von mehr als 2 % konsumiert worden, im letzten Jahr keine von mehr als 4 %.

#### Injizierender Substanzkonsum.

Anabole Steroide wurden von 1,9 % der Befragten etwas weniger injiziert als Substanzen, um high zu werden (2,6 %). Ein Fünftel derjenigen, die jemals injiziert hatten, hatte dies mit einer gebrauchten Nadel oder Spritze getan (0,5 % aller Befragten).

#### Kombination von Sex mit Substanzkonsum.

15 % hatten jemals Chemsex^2^, 2 Drittel davon in den letzten 12 Monaten. Chemsex in einer Gruppe war häufig, aber meistens wurden Chemsex-Substanzen zusammen mit *einem* Partner verwendet.

### Bedarfe

Bedarfe in Bezug auf sexuelle Gesundheit sind definiert als die Fähigkeiten, Möglichkeiten und Motivationen, sich auf gesundheitsbezogenes sexuelles Verhalten einzulassen, sowohl im positiven als auch im negativen Sinn. Ein zentrales Forschungsziel von EMIS war es, sexuelle Gesundheitsbedarfe zu identifizieren, die häufig nicht erfüllt werden, damit diese Bedarfe bei Interventionen priorisiert werden können.

#### Soziale Unterstützung.

Bei Verwendung von 2 Unterskalen der Skala für soziale Unterstützung („social provisions scale“ – SPS) mangelte es bei 7 % an einer verlässlichen Unterstützung und 9 % waren sozial wenig integriert.

#### Internalisierte Homonegativität.

12 % zeigten Hinweise auf eine internalisierte Homonegativität, d. h., die Antworten dieser Männer deuten hin auf eine negative Einstellung gegenüber Homosexualität im Allgemeinen und gegenüber ihrer eigenen sexuellen Orientierung.

#### Safer Sex.

Insgesamt 29 % wussten nicht, dass die meisten sexuell übertragbaren Krankheiten leichter zu übertragen sind als HIV; 22 % hatten im letzten Jahr kondomlosen Sex, nur weil sie keinen Zugang zu Kondomen hatten. 6 % hatten Sex, der nicht so sicher wie gewünscht war, und 7 % fanden es schwierig, unerwünschten Sex abzulehnen.

#### Hepatitisimpfung.

32 % der Befragungsteilnehmer wussten nicht, dass das Gesundheitssystem für MSM eine Impfung gegen Hepatitis A und B empfiehlt.

#### PEP.

40 % der Befragungsteilnehmer hatten noch nichts von PEP gehört.

#### PrEP.

36 % der Stichprobe hatten noch nichts von PrEP gehört.

#### HIV-Tests und -Behandlungen.

40 % wussten nicht, dass Menschen, die eine wirksame Behandlung erhalten, HIV nicht übertragen können, und 3 % waren sich ihres HIV-Status „nicht sicher“.

### Interventionen

Interventionen können sich positiv (Abdeckung von Bedarfen) oder negativ (Untergrabung von Bedarfen und Erzeugung eines nicht gedeckten Bedarfs) auswirken. Positive Interventionen umfassen Bildung, Gesundheits- und Sozialdienste sowie eine Vielzahl von Möglichkeiten, wie sich Communitymitglieder gegenseitig helfen. Negative Interventionen umfassen homophobe Gesetzgebung, Ausgrenzung und homophoben Missbrauch.

#### Homophober Missbrauch, Einschüchterung, Beleidigungen und Gewalt.

In den letzten 12 Monaten wurden 1,5 % der Teilnehmer körperlich angegriffen, 20 % wurden beleidigt und 25 % waren eingeschüchtert worden, weil jemand wusste oder vermutete, dass sie sich zu Männern hingezogen fühlten.

#### Zugang zu kostenlosen Kondomen.

32 % gaben an, in den letzten 12 Monaten kostenlos Kondome von Organisationen, Gesundheitseinrichtungen, in Bars oder in Saunen erhalten zu haben.

#### HIV-/STI-Aufklärungsangebote.

87 % hatten im letzten Jahr MSM-spezifische Informationen über HIV oder STI gesehen, mehr als die Hälfte (51 %) auch in den letzten 4 Wochen.

#### HIV-Testung.

46 % aller nicht mit HIV diagnostizierten Teilnehmer und 62 % aller jemals auf HIV getesteten und nicht mit HIV diagnostizierten Teilnehmer hatten in den letzten 12 Monaten ein HIV-Testergebnis erhalten, am häufigsten in Haus- oder Facharztpraxen. In Gesundheitsämtern oder communitybasierten Testangeboten hatte sich ein weiteres Drittel testen lassen.

#### HIV-Behandlungskaskade.

Unsere Daten ermöglichen die Konstruktion der letzten 4 Stufen der HIV-Behandlungskaskade, d. h. jemals Zugang zu medizinischer Betreuung, aktuell in medizinischer Betreuung, unter antiretroviraler Therapie, nicht nachweisbare Viruslast. Unter den mit HIV diagnostizierten Teilnehmern wurden das zweite und dritte der 3 90-90-90-Ziele^3^ der Weltgesundheitsorganisation erreicht.

#### STI-Testangebote.

38 % waren in den letzten 12 Monaten auf andere STI als HIV getestet worden. Ein vollständiges STI-Screening, definiert als HIV-Test, Bluttest auf STI, Urinprobe oder Harnröhrenabstrich und Analabstrich, wurde im letzten Jahr von 8 % der nicht mit HIV diagnostizierten Teilnehmer berichtet.

#### Partnerbenachrichtigung.

Die Mehrheit der Teilnehmer (78 %), bei denen in den letzten 12 Monaten entweder eine Syphilis oder eine Gonorrhö diagnostiziert worden war, hatte zumindest einige ihrer Sexualpartner darüber informiert, dass sie sich vorsichtshalber testen oder behandeln lassen sollten.

#### Hepatitisimpfung.

Mehr als die Hälfte hatte jemals eine Hepatitisimpfung erhalten. Jeweils 57 % gaben an, eine vollständige Impfung gegen Hepatitis A und gegen Hepatitis B erhalten zu haben.

### Gesundheitliche Ungleichheiten

**Figure 1:**
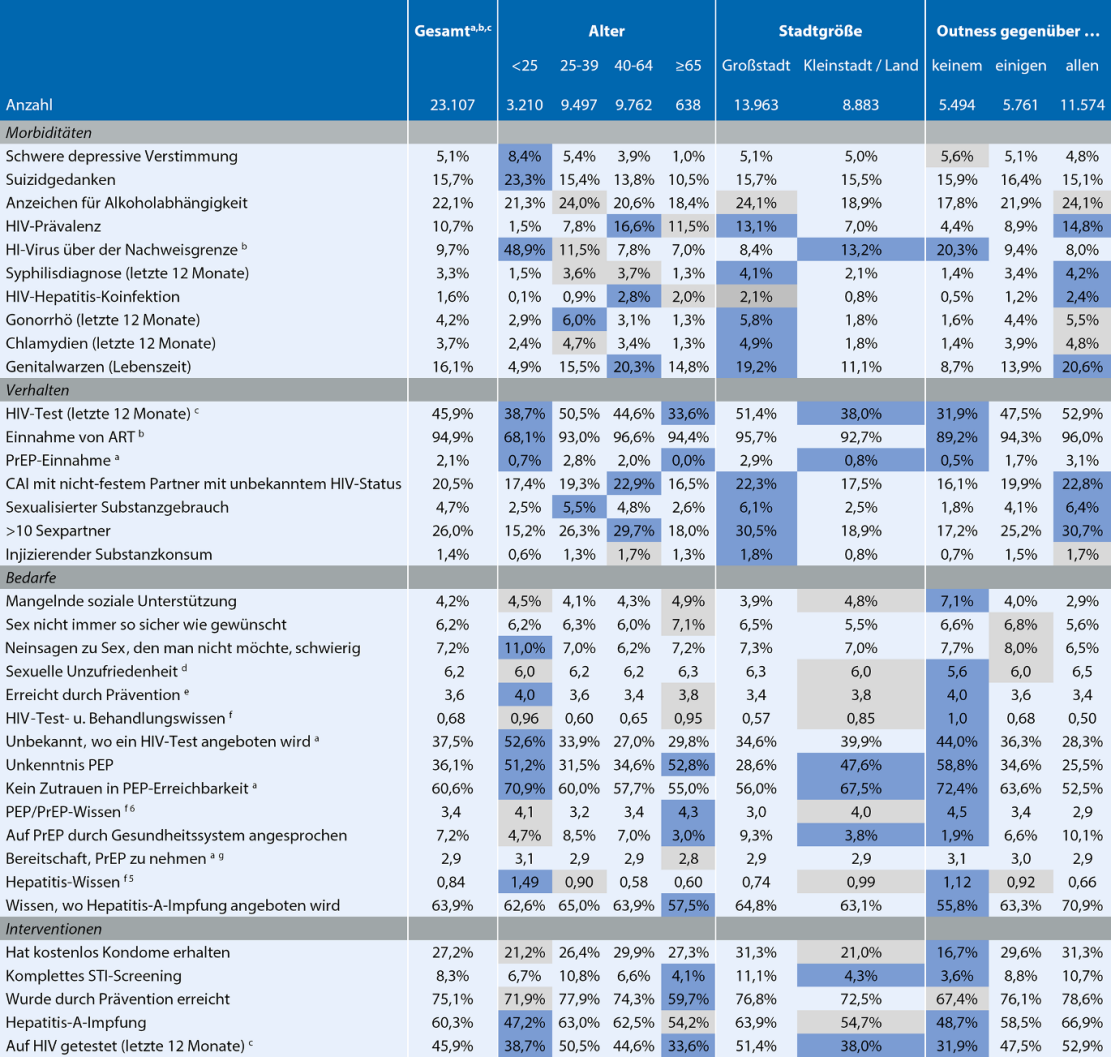

Abb. 1 zeigt das Ausmaß von Erkrankungen, Verhalten, ungedeckten Präventionsbedarfen und Interventionen in verschiedenen Untergruppen von MSM, die Ziele für Public-Health-Maßnahmen sein können. Zu diesem Zweck betrachten wir, wie sich die oben beschriebenen Indikatoren zwischen den genannten Hauptzielgruppen unterscheiden.

**Figure 2:**
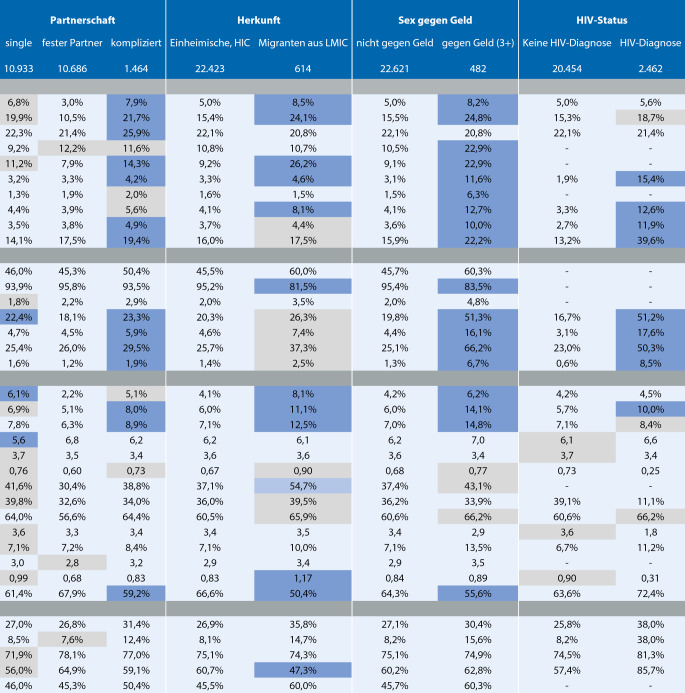

Zusammenfassend betrachten wir Indikatoren für 4 wichtige demografische Merkmale (Alter; Outness; Beziehungsstatus; Ortsgröße) und 3 MSM-Minderheitsgruppen (MSM, die aus außereuropäischen Ländern mit niedrigen und mittleren Einkommen stammen; MSM, die sexuelle Dienstleistungen anboten, sowie MSM mit einer HIV-Diagnose).

## Diskussion

EMIS-2017 ist es gelungen, ein breites, wenngleich nicht repräsentatives Spektrum von in Deutschland lebenden MSM abzubilden. Männer, die sich offen zu ihrer sexuellen Orientierung bekennen, und sexuell aktivere Männer sind wahrscheinlich überrepräsentiert. Wie bei fast allen nicht repräsentativen Umfragen sind bildungsferne Personen im Untersuchungssample wahrscheinlich unterrepräsentiert.

Von großer Bedeutung für die Präventionsarbeit für und in der Gruppe der MSM ist die höhere Verbreitung von psychischen Belastungen und STI. Bei den psychischen Problemen stehen vor allem für die jüngeren Altersgruppen depressive Verstimmung und Suizidalität – oft im Kontext der sozialen Diskriminierung sexueller Minoritäten – im Vordergrund, für die übrigen Altersgruppen gewinnen Alkoholabhängigkeit und der Ge- und Missbrauch psychoaktiver Substanzen – oft im Kontext mit Sex – an Bedeutung.

Die psychischen Belastungen unterscheiden sich für homo- und bisexuelle Männer quantitativ, weniger qualitativ von denen in der männlichen Allgemeinbevölkerung. Es könnten bestehende Behandlungs- und Beratungsangebote für die Situation homo- und bisexueller Männer sensibilisiert werden. Ein adäquateres Diversitätsmanagement im Umgang mit Klient:innen ist aber im Grunde eine Aufgabe, der sich alle medizinischen Einrichtungen stellen müssen.

Von STI sind MSM deutlich stärker betroffen als die heterosexuelle Mehrheitsbevölkerung. Besondere Probleme und Herausforderungen bezüglich der Diagnose ergeben sich daraus, dass viele dieser Infektionen keine oder nur untypische Beschwerden verursachen. Die Abklärung der Ursache von Beschwerden entfällt daher häufig als primärer Anlass für eine Diagnostik und wird ersetzt durch eine auf erhöhtem individuellen und kollektiven Risiko basierende Risikoeinschätzung. In der deutschen EMIS-Stichprobe sind es 46 % der Teilnehmer, die ein HIV-Screening in den letzten 12 Monaten angaben, das liegt 9 % unter dem Durchschnittswert aller EU-Länder [20]. Besonders hoch mit über 60 % liegt der Anteil der Nichtgescreenten bei jüngeren und älteren MSM, bei MSM, die nicht so offen bezüglich ihrer sexuellen Orientierung sind, und in ländlichen Regionen. Das weist auf die Schwierigkeiten hin, überall ein wohnortnahes Testangebot zu finden, bei dem die Vertraulichkeit sichergestellt ist und wo es den Betroffenen leichtfällt, sich zu outen und/oder nach einem HIV-Test zu fragen. Vergleichbare Probleme gibt es bei den Impfungen gegen Hepatitis A und B, die für MSM in Deutschland empfohlen werden. Während ansonsten fast 60 % der Befragten eine Impfung berichten, liegt der Anteil bei jungen MSM (< 25 Jahre), Männern, deren soziales Umfeld bezüglich ihrer sexuellen Orientierung nicht informiert ist, und Männern, die aus ärmeren außereuropäischen Ländern stammen, bei unter 50 %.

Noch komplexer wird es beim Screening auf Gonokokken und Chlamydien. Es gibt zwar breite Evidenz dafür, dass asymptomatische Infektionen mit diesen beiden Erregern bei MSM häufig sind, vor allem rektal und pharyngeal, jedoch sieht das kassenärztliche Abrechnungssystem ein Screening mittels Polymerasekettenreaktion (PCR) nicht vor. Trotzdem wird ein solches Screening in den letzten Jahren international und auch in Deutschland zunehmend propagiert und in einem Graubereich zumindest bei Männern, die wegen HIV in Behandlung sind oder eine medikamentöse PrEP erhalten, auch über die Krankenkassen finanziert. Allerdings ist wissenschaftlich nicht geklärt, ob ein solches Screening medizinisch und epidemiologisch sinnvoll ist, da asymptomatische Infektionen mit diesen Erregern in aller Regel spontan nach einigen Wochen ausheilen und Langzeitkomplikationen bei nicht erfolgter Behandlung nicht bekannt sind. Die Frage, ob ein auf die sexuellen Praktiken bei MSM abgestimmtes Screening auf Gonokokken und Chlamydien erstrebenswert ist, kann daher derzeit nicht evidenzbasiert beantwortet werden [28, 29].

Die Versorgung von MSM mit HIV-Diagnose mit antiretroviraler Therapie ist in Deutschland sehr gut. Knapp 95 % erhalten aktuell eine ART und auch der Therapiestart nach einer HIV-Diagnose findet nach entsprechenden Leitlinienanpassungen in den letzten Jahren deutlich früher statt. Das bedeutet auch, dass von diesen diagnostizierten und in der Regel erfolgreich therapierten Personen kein HIV-Ansteckungsrisiko mehr ausgeht. Das Wissen um fehlende Ansteckungsfähigkeit bei erfolgreicher Therapie ist selbst bei den MSM noch nicht ausreichend verbreitet. Der präventive Gebrauch antiretroviraler Medikamente etwa im Rahmen einer PEP oder PrEP ist ebenfalls ausbaufähig. Zwar wurden die Daten zur PrEP Ende 2017 erhoben und sind inzwischen veraltet, u. a. weil die HIV-PrEP seit September 2019 auf Kosten der Gesetzlichen Krankenversicherung verschrieben werden kann, trotzdem weist eine (zur Publikation eingereichte) PrEP-Bedarfsanalyse für Deutschland mit Stand Mitte 2020 auf erhebliche regionale Ungleichheiten beim Zugang zu PrEP hin. EMIS-2017 zeigt auch, dass zumindest zum damaligen Zeitpunkt ein erheblicher Informationsbedarf zu PEP und PrEP bestand: Nahezu 40 % der Befragten hatten zum damaligen Zeitpunkt von diesen Präventionsmöglichkeiten noch nicht gehört.

Der Konsum von illegalen und psychoaktiven Substanzen ist bei MSM verbreitet. Am weitesten verbreitet jedoch sind – wie in der Allgemeinbevölkerung – Alkohol und Tabakprodukte. Insgesamt liegt aber das ganze Substanzkonsumniveau in der EMIS-Stichprobe höher als in der Allgemeinbevölkerung [30]. Das gilt auch für den Konsum nicht legaler Substanzen wie Cannabis, Ecstasy, Kokain und Amphetamine. Dazu kommt der Gebrauch von erektionserhaltenden und rezeptiven Analverkehr erleichternden Substanzen. Ein Phänomen, das in den letzten Jahren zunehmend an Aufmerksamkeit gewonnen hat, ist Chemsex. Unter Chemsex wird Sex unter dem Einfluss von synthetischen Drogen („Chems“) verstanden [17, 31]. Verwendet werden häufig Substanzen wie GHB/GBL, Mephedron, Ketamin und Crystal Meth. Das Phänomen ist weltweit in der Schwulenszene verbreitet.

Längst nicht jeder Substanzkonsum ist per se unter Präventionsaspekten problematisch. Problematisch kann er dann werden, wenn er Suchtcharakter annimmt, zu gesundheitlichen, psychischen und sozialen Störungen führt oder beiträgt. EMIS-2017 zeigt einen hohen und mit zunehmendem Alter ansteigenden Anteil von problematischem Alkoholkonsum, der in Großstädten ausgeprägter ist als in ländlichen Regionen. Chemsex findet in Großstädten ebenfalls häufiger statt. Mehrheitlich werden Chemsex-Substanzen zwar in dyadischen sexuellen Begegnungen verwendet, aber auch häufig in Gruppensexsettings wie bei öffentlichen und privaten Sexpartys. Die Teilnehmer solcher Partys nehmen überproportional häufig antiretrovirale Medikamente zur Therapie oder Prophylaxe und verwenden daher seltener Kondome. Auch wenn das HIV-Übertragungsrisiko in solchen Settings daher nicht so hoch ist, wie man annehmen könnte, können andere bakterielle und virale STI hier leicht übertragen werden.

Gesundheitlich besonders problematisch sind Infektionen mit Syphilis und Hepatitis C. Das Hepatitis-C-Risiko steigt weiter an, wenn Chemsex-Substanzen injiziert und Utensilien geteilt werden. Auch wenn die EMIS-Daten nahelegen, dass problematischer Chemsex-Gebrauch nur eine kleine Minderheit betrifft, sind Beratungs‑, Hilfs- und bei Bedarf auch therapeutische Angebote notwendig. Für die Entwicklung von individuellem Problembewusstsein und die Akzeptanz von Hilfsangeboten ist zu berücksichtigen, dass die Ziele des Gebrauchs von Chemsex-Substanzen der Abbau der Hemmungen und die Steigerung der sexuellen Lust sind. Diese Ziele werden subjektiv oft zunächst auch erreicht. Erst im weiteren Verlauf werden u. U. negative soziale, psychische und gesundheitliche Folgen erkennbar.

## Fazit

Niedrigschwellige Online-Surveys wie EMIS-2017 können wertvolle Informationen zu Männern, die Sex mit Männern haben, erheben, die mit anderen Instrumenten kaum oder nur mit deutlich höherem personellen und finanziellen Aufwand gewonnen werden könnten. Gesundheitliche Belastungen, Präventionsbedarfe und Passgenauigkeit von Interventionen lassen sich – auch für Untergruppen – gut identifizieren und weitere Forschungsbedarfe erkennen. Änderungen von Verhalten, die z. T. auch Anpassungen bei den Präventionsbotschaften und -strategien erfordern oder durch diese unterstützt wurden, können durch wiederholte Befragungen erkannt werden.

## Supplementary Information





## Footnotes

1 Allerdings berichteten nur 20 % der Teilnehmer von kondomlosem Analverkehr mit einem Partner mit unbekanntem HIV-Serostatus, 63 % berichteten *keinen* kondomlosen Analverkehr mit einem nicht festen Partner und 43 % berichteten kondomlosen Analverkehr weder mit einem festen, noch mit einem nicht festen Partner in den letzten 12 Monaten.

2 Unter Chemsex wird Sex unter dem Einfluss von synthetischen Drogen („Chems“) verstanden. Verwendet werden häufig Substanzen wie GHB/GBL, Mephedron, Ketamin und Crystal Meth.

3 Mindestens 90 % der HIV-Infizierten sollten diagnostiziert sein (Ziel 1), mindestens 90 % der Menschen mit HIV-Diagnose sollten eine antiretrovirale Therapie erhalten (Ziel 2) und mindestens 90 % der Therapierten sollten eine Viruslast unter der Nachweisgrenze erreichen (Ziel 3).
