# Supplementary material for: The European MSM Internet Survey as a basis for prevention work in Germany for men who have sex with men
Source: Bundesgesundheitsblatt Gesundheitsforschung Gesundheitsschutz. 2021 Oct 13;64(11):1430–9. [Article in German] doi: 10.1007/s00103-021-03429-3 (PMC8550191; doi:10.1007/s00103-021-03429-3)
Supplement: Supplementary file 1 [file 103_2021_3429_MOESM1_ESM.pdf]

Bundesgesundheitsblatt 11/2021

Onlinematerial zum Beitrag:

Der Europäische MSM Internet Survey als Grundlage für die Präventionsarbeit in Deutschland für Männer, die Sex mit Männern haben

Ulrich Marcus[1], Susanne B. Schink[1]
[1]Robert Koch-Institut, Abt. Infektionsepidemiologie, Berlin, Deutschland

Korrespondenzadresse
Dr. Ulrich Marcus
Robert Koch-Institut
Seestraße 10
13353 Berlin
Deutschland
MarcusU@rki.de

Inhalt:

EMIS-Fragenkatalog, geordnet nach Themengebieten

EMIS-Fragenkatalog, geordnet nach Themengebieten

Antwortmöglichkeiten:

- Wissensfragen: Das wusste ich bereits / Darüber war ich mir nicht sicher / Das wusste ich noch nicht / Ich verstehe das nicht / Ich glaube nicht, dass das stimmt.
- Zustimmungs- bzw. Ablehnungsfragen: zustimmen – nicht zustimmen
- Likert-Skala

1.0 GESUNDHEITSZUSTAND

1.1 ZUFRIEDENES SEXLEBEN

Wie zufrieden sind Sie mit Ihrem Sexleben?

1.2 MENTALE GESUNDHEIT

Wie oft fühlten Sie sich in den letzten zwei Wochen durch die folgenden Beschwerden beeinträchtigt?

Gefühle der Nervosität, Ängstlichkeit oder Anspannung
Nicht in der Lage sein, Sorgen zu stoppen oder zu kontrollieren
Wenig Interesse oder Freude an Ihren Aktivitäten
Niedergeschlagenheit, Bedrücktheit oder Hoffnungslosigkeit
Gedanken, dass Sie lieber tot wären oder sich Leid zufügen möchten

1.3 DIAGNOSEN SEXUELL ÜBERTRAGENER INFEKTIONEN

Wann wurde bei Ihnen zuletzt Syphilis diagnostiziert?
Wann wurde bei Ihnen zuletzt Gonorrhö diagnostiziert?
Wann wurde bei Ihnen zuletzt Chlamydien diagnostiziert?
Wann wurden bei Ihnen ZUERST Anal- oder Genitalwarzen diagnostiziert?
Wann wurde bei Ihnen ZUERST Anal- oder Genitalherpes diagnostiziert?
Wann wurde bei Ihnen ZUERST Hepatitis C diagnostiziert?
Wie ist Ihr aktueller Hepatitis-C-Status?
[siehe Abschnitt 2.3.2 für Hepatitis B]

1.4 DIAGNOSE VON HIV

In welchem Jahr wurde bei Ihnen erstmals HIV diagnostiziert?
Wurde bei Ihnen in den letzten 12 Monaten erstmals HIV diagnostiziert?

1.5 SCHNELLE HIV-DIAGNOSE

Wie hoch war Ihre CD4-Zahl, als bei Ihnen zum ersten Mal HIV diagnostiziert wurde?

1.6 HIV-VIRALE UNTERDRÜCKUNG

Was war das Ergebnis der Viruslastbestimmung bei Ihrer letzten Kontrolluntersuchung?

2.1 VERHALTEN: SEX

2.1.1 ERSTER UND LETZTER SEX MIT MÄNNERN

Wie alt waren Sie, als Sie zum ersten Mal Sex mit einem Mann/Jungen hatten oder ein Mann/Junge Sex mit Ihnen hatte?
Wie alt waren Sie, als Sie zum ersten Mal Analverkehr mit einem Mann/Jungen hatten?
Wann hatten Sie zuletzt Analverkehr mit einem Mann (entweder mit oder ohne Kondom)?
Hatten Sie bei der letzten Gelegenheit des Analverkehrs ungeschützten Analverkehr (d.h. ohne Kondom)?
Wann hatten Sie zuletzt ungeschützten Analverkehr mit einem Mann (d.h. ohne Kondom)?

2.1.2 SEX MIT FESTEN MÄNNLICHEN PARTNERN IN DEN LETZTEN 12 MONATEN

Hatten Sie in den letzten 12 Monaten Sex mit einem festen männlichen Partner?
Mit wie vielen verschiedenen festen männlichen Partnern haben Sie in den letzten 12 Monaten Sex gehabt?
Mit wie vielen festen männlichen Partnern hatten Sie in den letzten 12 Monaten Analverkehr?
Mit wie vielen festen männlichen Partnern hatten Sie in den letzten 12 Monaten Analverkehr ohne Kondom?
Wie oft hatten Sie in den letzten 12 Monaten Analverkehr mit Ihrem festen männlichen Partner?
Wie oft wurden in den letzten 12 Monaten Kondome für den Analverkehr mit Ihrem festen männlichen Partner verwendet?
Haben Sie beim letzten Analverkehr mit Ihrem festen männlichen Partner ein Kondom benutzt?
Haben Sie und dieser feste männliche Partner den gleichen HIV-Status?

2.1.3 SEX MIT NICHT FESTEN MÄNNLICHEN PARTNERN IN DEN LETZTEN 12 MONATEN

Hatten Sie in den letzten 12 Monaten Sex mit einem nicht-festen männlichen Partner?
Mit wie vielen verschiedenen nicht-festen männlichen Partnern hatten Sie in den letzten 12 Monaten Sex?
Mit wie vielen nicht-festen Partnern hatten Sie in den letzten 12 Monaten Analverkehr?
Mit wie vielen nicht-festen männlichen Partnern hatten Sie in den letzten 12 Monaten Analverkehr ohne Kondom?
Hatten Sie in den letzten 12 Monaten Analverkehr mit einem nicht-festen männlichen Sexpartner?
Mit welchem Anteil Ihrer nicht-festen männlichen Sexualpartner hatten Sie in den letzten 12 Monaten Analverkehr?
Wie oft wurden in den letzten 12 Monaten Kondome verwendet, wenn Sie Analverkehr mit nicht-festen männlichen Partnern hatten?
Hatten Sie ungeschützten Analverkehr mit einem nicht-festen Partner, von dem Sie zu diesem Zeitpunkt wussten, dass er HIV-positiv ist?
Hatten Sie ungeschützten Analverkehr mit einem nicht-festen Partner, von dem Sie zu diesem Zeitpunkt wussten, dass er HIV-negativ war?
Hatten Sie ungeschützten Analverkehr mit einem nicht-festen Partner, dessen HIV-Status Sie zu diesem Zeitpunkt nicht kannten oder über den Sie nicht nachdachten?

2.1.4 CHARAKTERISTIKA DES LETZTEN SEXUELLEN KONTAKTS MIT EINEM NICHT FESTEN MÄNNLICHEN PARTNER

Wie viele Personen waren beteiligt?
Wo hat der Sex stattgefunden?
Wo haben Sie ihn (Ihre(n) letzten Partner) kennengelernt?
Hatten Sie schon einmal Sex mit ihm (bei einer anderen Gelegenheit)?
Was haben Sie ihm über Ihren HIV-Status vor oder beim Sex erzählt?
Was haben Sie zu Ihrem PrEP-Gebrauch mitgeteilt? Was hat er zu seinem PrEP-Gebrauch mitgeteilt?
Was haben Sie ihm über Ihre Viruslast mitgeteilt? Was hat er Ihnen über seine Viruslast mitgeteilt?
Was wussten oder dachten Sie über seinen HIV-Status, bevor Sie Sex hatten?
Hatten Sie bei dieser Gelegenheit Analverkehr?
Hat er ein Kondom benutzt, als er beim Analverkehr aktiv war (als er Sie gefickt hat)?
Hat er in Ihr Rektum (Arsch) ejakuliert?
Haben Sie ein Kondom benutzt, als Sie beim Analverkehr "aktiv" waren?
Haben Sie in sein Rektum (Arsch) ejakuliert?
Wie würden Sie diese sexuelle Begegnung auf einer Skala von 1 (am schlechtesten) bis 10 (am besten) bewerten?

2.1.5 SEX MIT FRAUEN IN DEN LETZTEN 12 MONATEN

Wann hatten Sie das letzte Mal Sex mit einer Frau?
Mit wie vielen (unterschiedlichen) Frauen haben Sie in den letzten 12 Monaten gefickt?
Wie oft haben Sie in den letzten 12 Monaten beim Vaginal- oder Analverkehr mit Frauen Kondome verwendet?

2.1.6 BETEILIGUNG AN KOMMERZIELLEM SEX

Wann haben Sie das letzte Mal einen Mann für Sex bezahlt? Unter Bezahlung verstehen wir den Austausch von Geld, Geschenken, oder Gefälligkeiten für Sex.
Wie oft haben Sie in den letzten 12 Monaten einen Mann für Sex bezahlt?
Wann wurden Sie das letzte Mal von einem Mann für Sex bezahlt? Unter Bezahlung verstehen wir den Austausch von Geld, Geschenken oder Gefälligkeiten für Sex.
Wie oft wurden Sie in den letzten 12 Monaten von einem Mann für Sex bezahlt?

2.2 VERHALTEN: DROGENKONSUM

2.2.1 AKTUALITÄT DES DROGENKONSUMS

Haben Sie jemals Anabolika (Testosteron) injiziert?
Haben Sie jemals eine andere Substanz als Anabolika oder Medikamente injiziert?
Wann haben Sie das letzte Mal Alkohol konsumiert?
Wann haben Sie das letzte Mal Tabakprodukte konsumiert?
Wann haben Sie das letzte Mal Poppers (Nitrit-Inhalationsmittel) konsumiert?
Wann haben Sie das letzte Mal Viagra®, Cialis®, Levitra® oder andere Substanzen konsumiert, die zur Aufrechterhaltung einer Erektion beitragen?
Wann haben Sie das letzte Mal Beruhigungsmittel (Valium®, Rivotril®, Rohypnol®) konsumiert?
Haben Sie jemals andere Freizeit- oder illegale Drogen genommen?

Wann haben Sie das letzte Mal Cannabis (Haschisch, Marihuana) konsumiert?
Wann haben Sie das letzte Mal Ecstasy konsumiert (E, XTC, MDMA)?
Wann haben Sie das letzte Mal Amphetamin konsumiert (Speed)?
Wann haben Sie das letzte Mal Crystal Meth (Kristall, Meth, Tina) konsumiert?
Wann haben Sie das letzte Mal Heroin oder verwandte Drogen (z.B. Fentanyl) konsumiert?
Wann haben Sie das letzte Mal Mephedron (4-MMC, Miau, Methylon) konsumiert?
Wann haben Sie das letzte Mal GHB/GBL (flüssiges Ecstasy) konsumiert?
Wann haben Sie das letzte Mal Ketamin (special K) konsumiert?
Wann haben Sie das letzte Mal LSD konsumiert?
Wann haben Sie das letzte Mal Kokain konsumiert?
Wann haben Sie das letzte Mal Crack-Kokain konsumiert?

2.2.2 KOMBINATION VON SEX UND DROGENKONSUM („CHEMSEX“)

Wann hatten Sie das letzte Mal nüchtern Sex, das heißt, vollständig unbeeinflusst von Alkohol oder anderen Drogen?
Wie oft hatten Sie in den letzten 12 Monaten Sex mit Männern unter dem Einfluss von Drogen oder Alkohol?
Wann haben Sie das letzte Mal stimulierende Substanzen konsumiert, um längeren oder intensiveren Sex zu haben?
Wann haben Sie das letzte Mal stimulierende Substanzen im Rahmen von Gruppensex (Sex mit mehr als einem Mann) genommen?
Wie viele Jahre kombinieren Sie schon stimulierende Substanzen und Sex mit mehreren Sexualpartnern?

2.3 VERHALTEN: NUTZUNG VON STI-VERHINDERNDEN KLINISCHEN DIENSTLEISTUNGEN

2.3.2 HEPATITIS-A und B-IMPFUNG

Wurde Ihnen jemals seitens eines Gesundheitsdienstes eine Hepatitis-Impfung angeboten?
Sind Sie gegen Hepatitis A geimpft?
Sind Sie gegen Hepatitis B geimpft?
Wissen Sie wo Sie sich gegen Hepatitis A impfen lassen können?
Wissen Sie wo Sie sich gegen Hepatitis B impfen lassen können?

2.3.3 HIV-TEST

Haben Sie jemals ein HIV-Testergebnis erhalten?
Wurde bei Ihnen jemals eine HIV-Infektion festgestellt?
Wurde Ihnen jemals seitens eines Gesundheitsdienstes (ärztliche Praxis, Krankenhaus, usw.) ein HIV-Test angeboten?
Wissen Sie, wo Sie sich auf HIV testen lassen können?
Wann wurden Sie das letzte Mal auf HIV getestet?
Wo haben Sie sich das letzte Mal auf HIV testen lassen?

2.3.4 ASYMPTOMATISCHE STI-TESTUNG

Haben Sie jemals einen Test auf andere sexuell übertragbare Infektionen (STIs) als HIV durchgeführt?
Wann hatten Sie zuletzt einen Test auf andere STIs als HIV?
Hatten Sie bei dieser Gelegenheit irgendwelche Symptome?

2.3.5 PROPHYLAXE NACH DER EXPOSITION

Wurden Sie jemals mit PEP behandelt?
Wurden Sie jemals mit PEP behandelt, bevor bei Ihnen HIV diagnostiziert wurde?

2.3.6 PROPHYLAXE VOR DER EXPOSITION

Haben Sie jemals versucht, die PrEP zu bekommen?
Haben Sie jemals die PrEP eingenommen?
Haben Sie mit einem Arzt/einer Ärztin gesprochen, bevor Sie mit der PrEP begonnen haben?

3.0 BEDÜRFNISSE (Möglichkeiten, Fähigkeiten und Motivationen für die sexuelle Gesundheit - die Grundlagen dafür)

3.1 VERTRAUEN IN DEN HIV-STATUS

Wie ist Ihrer Meinung nach Ihr aktueller HIV-Status (unabhängig davon, ob Sie jemals auf HIV getestet haben oder nicht)?

3.2 SEXUELLE SELBSTWIRKSAMKEIT

Der Sex, den ich habe, ist immer so sicher, wie ich möchte.
Es fällt mir leicht, "nein" zu sagen zu Sex, den ich nicht möchte.

3.3 WISSEN ÜBER HIV, TESTUNG UND BEHANDLUNG

Wann haben Sie zuletzt Informationen über HIV oder andere sexuell übertragene Infektionen gesehen oder gehört, die sich speziell an Männer richten, die Sex mit Männern haben?

AIDS wird durch ein Virus verursacht, das HIV heißt.
Man kann nicht am äußeren Erscheinungsbild erkennen, ob jemand HIV hat oder nicht.
Es gibt einen medizinischen Test, der zeigen kann, ob man HIV hat oder nicht.
Wenn sich jemand mit HIV infiziert, kann es einige Wochen dauern, bis man es in einem Test nachweisen kann.
Es gibt derzeit kein Heilmittel gegen HIV.
Eine HIV-Infektion ist heute mit Medikamenten gut behandelbar, so dass die gesundheitlichen Folgen deutlich geringer sind.
Wenn ein HIV-Infizierter eine wirksame Therapie erhält und die Viruslast nicht nachweisbar ist ("undetectable"), kann er HIV sexuell nicht weitergeben.

3.4 WISSEN ÜBER DIE HIV-/STI-ÜBERTRAGUNG

HIV kann nicht durch Küssen weitergegeben werden, da Speichel HIV nicht überträgt. Man kann sich beim "aktiven" ungeschützten Ficken mit einem/r infizierten Partner/in über seinen Penis mit HIV anstecken, auch wenn man nicht ejakuliert.
Man kann sich über seinen Enddarm (oder seine Vagina) beim "passiven" ungeschützten Ficken mit einem infizierten Partner mit HIV anstecken.
Die meisten STI können leichter weitergegeben werden als HIV.
STI sind häufig symptomlos, und man kann sich angesteckt haben ohne es zu merken.
Die richtige Anwendung von Kondomen beim Ficken reduziert die Wahrscheinlichkeit, sich bzw. andere mit STI (oder HIV) anzustecken.

3.5 WISSEN ÜBER PEP

Haben Sie schon mal von PEP gehört?

Die Postexpositionsprophylaxe (PEP) versucht, eine HIV-Infektion zu verhindern, nachdem man dem Virus ausgesetzt war (z.B. nach ungeschütztem Analverkehr).
Eine PEP ist eine Behandlung gegen HIV, bei der Medikamente für den Zeitraum eines Monats eingenommen werden.
Eine PEP sollte so schnell wie möglich nach einem Risikokontakt begonnen werden, am besten innerhalb weniger Stunden.

3.6 WISSEN ÜBER PREP

Haben Sie schon mal von der PrEP gehört?
Bei der HIV-Prä-Expositionsprophylaxe (PrEP) nimmt eine HIV-negative Person Tabletten vor und nach dem Sex, um sich vor HIV zu schützen.
Die PrEP kann als tägliche Tablette eingenommen werden, vor allem wenn man nicht so genau im Voraus weiß, wann man das nächste Mal Sex hat.
Bei geplanten Sexkontakten kann die PrEP auch in Form von 2 Tabletten 24 Stunden vor dem Sex, und jeweils einer Tablette 24 und 48 nach der ersten Dosis eingenommen werden.

3.7 ZUGANG ZU KONDOMEN

Wann hatten Sie das letzte Mal ungeschützten Analverkehr, nur weil Sie kein Kondom hatten?

3.8 ZUGANG ZUR HIV/STI-TESTUNG

Wie sicher sind Sie, dass Sie einen HIV-Test erhalten könnten, wenn Sie einen wollten?
Wie sicher sind Sie, dass Sie einen weiteren HIV-Test erhalten könnten, wenn Sie einen wollten?

3.9 ZUGANG ZU PEP

Wie sicher sind Sie, dass Sie eine PEP erhalten, wenn Sie eine brauchen?

3.10 ZUGANG ZU PREP

Haben Sie in Ihrem Wohnland jemals ein Rezept für die PrEP erhalten (Verschreibung)?
Wo haben Sie ein PrEP-Rezept erhalten?
Wo haben Sie die Tabletten für Ihre PrEP erhalten?
Wenn die PrEP für Sie verfügbar und bezahlbar wäre, wie wahrscheinlich wäre es, dass Sie die PrEP nehmen?

3.11 ZUGANG ZUR HIV-BEHANDLUNG

<GRÜNDE FÜR NIE EINE HIV-BEHANDLUNG ERHALTEN>

Mein Arzt sagt, dass ich derzeit keine antiretrovirale Behandlung benötige.
Um die Nebenwirkungen zu vermeiden.
Ich halte es nicht für notwendig.
Ich fürchte, die Leute werden es bemerken.
Ich möchte nicht jeden Tag an HIV erinnert werden.
Die Behandlung ist in dem Land, in dem ich lebe, nicht verfügbar.
Ich kann mir die Behandlung nicht leisten.
Anderer Grund [warum niemals eine antiretrovirale Behandlung eingenommen wird]

<GRÜNDE FÜR DIE BEENDIGUNG DER HIV-BEHANDLUNG>

Mein Arzt sagt, dass ich derzeit keine antiretrovirale Behandlung benötige
Um die Nebenwirkungen zu vermeiden
Ich halte es nicht für notwendig
Ich fürchte, die Leute werden es bemerken
Ich möchte nicht jeden Tag an HIV erinnert werden
Die Behandlung ist in dem Land, in dem ich lebe, nicht mehr verfügbar
Ich kann mir die Behandlung nicht mehr leisten
Anderer Grund [für den Abbruch der antiretroviralen Behandlung]?

3.12 DROGENVERWENDUNG

Ich mache mir Sorgen über meinen Drogenkonsum.
Haben Sie jemals einen Arzt/eine Ärztin oder eine Klinik aufgesucht, weil Sie sich Sorgen hinsichtlich Ihres Drogenkonsums (ausser Alkohol/Nikotin) gemacht haben?
Haben Sie jemals eine Drogen-Selbsthilfegruppe oder eine Drogenberatungsstelle aufgesucht, weil Sie sich Sorgen hinsichtlich Ihres Drogenkonsums gemacht haben (abgesehen von Alkohol oder Nikotinentwöhnung)?
Haben Sie schon einmal versucht, Ihren Alkoholkonsum zu reduzieren?
Haben andere Menschen Sie schon mal wegen Ihres Alkoholkonsums kritisiert?
Haben Sie sich wegen Ihres Alkoholkonsums schon mal schlecht oder schuldig gefühlt?
Kam es vor, dass Sie nach dem Aufwachen Alkohol getrunken haben, um Ihre Nerven zu beruhigen oder Ihren Kater loszuwerden?
Haben Sie jemals einen Arzt/eine Ärztin oder eine Klinik aufgesucht, weil Sie sich Sorgen hinsichtlich ihres Alkoholkonsums gemacht haben?

3.13 SOZIALE UNTERSTÜTZUNG

Es gibt Menschen, auf die ich im Notfall zählen kann.
Es gibt niemanden, der meine Belange und Interessen teilt.
Es gibt Menschen, die die gleichen gesellschaftlichen Aktivitäten mögen wie ich.
Es gibt niemanden, auf dessen Hilfe ich mich verlassen kann, wenn ich sie wirklich brauche.
Es gibt niemanden, der gerne die gleichen Dinge unternimmt wie ich.
Es gibt Menschen, auf deren Hilfe ich mich verlassen kann, wenn ich sie brauche.
Ich fühle mich als Teil einer Gruppe von Menschen, die meine Ansichten und Werte teilen.
Falls etwas in meinem Leben schiefgeht, würde mir niemand helfen.

3.14 MENTALE GESUNDHEIT

• Internalisierte Homonegativitätsskala

- Ich fühle mich in Schwulenkneipen/-bars wohl.
- Ich fühle mich in Gegenwart von offen schwulen Männern unwohl.
- Es macht mir nichts aus, wenn ich in der Öffentlichkeit mit einer offensichtlich schwulen Person gesehen werde.
- Es macht mir nichts aus, in der Öffentlichkeit über Homosexualität zu sprechen.
- Ich fühle mich wohl als homosexueller Mann.
- Homosexualität ist für mich moralisch akzeptabel.
- Ich würde meine sexuelle Orientierung nicht ändern, selbst wenn ich es könnte.

4.0 INTERVENTIONEN

4.1 INFORMATIONEN ÜBER HIV & STIs

Wann haben Sie das letzte Mal Informationen über HIV oder sexuell übertragbare Krankheiten speziell für Männer die Sex mit Männern haben gesehen oder gehört?

4.2 HIV-TESTANGEBOTE (NEGATIV GETESTET) - ABDECKUNG UND ANNAHME

Wo haben Sie sich das letzte Mal auf HIV testen lassen?
Wie zufrieden waren Sie mit der Unterstützung und den Informationen, die Sie bei Ihrem letzten HIV-Test erhalten haben?

4.3 HIV-TEST- UND -BEHANDLUNGSANGEBOTE (POSITIV GETESTET) - ABDECKUNG UND ANNAHME

Wo erfolgte Ihre HIV-Diagnose? Wie zufrieden waren Sie mit der Unterstützung und den Informationen, die Sie bei Ihrer HIV-Diagnose erhalten haben?
Wann haben Sie das letzte Mal einen Arzt/eine Ärztin zur Verlaufskontrolle Ihrer HIV-Infektion aufgesucht?
Haben Sie jemals eine gegen HIV gerichtete Behandlung mit Medikamenten (ART, HAART, Kombinationstherapie) bekommen? Warum nicht?
Wie lange hat es nach Ihrer HIV-Diagnose gedauert, bis Sie mit einer HIV-Therapie begonnen haben?
Erhalten Sie derzeit eine gegen HIV gerichtete medikamentöse Behandlung?

4.4 STI-TESTUNG - ABDECKUNG

Haben Sie in den letzten 12 Monaten im Rahmen eines STI-Tests eine Blutprobe zur Verfügung gestellt?
Haben Sie in den letzten 12 Monaten im Rahmen eines STI-Tests eine Urinprobe bereitgestellt?
Wurde Ihr Penis in den letzten 12 Monaten im Rahmen eines STI-Tests untersucht?
Wurde in den letzten 12 Monaten im Rahmen eines STI-Tests etwas in die Öffnung Ihres Penis (Harnröhrenabstrich) eingeführt?
Wurde Ihr Anus in den letzten 12 Monaten im Rahmen eines STI-Tests untersucht?
Wurde in den letzten 12 Monaten im Rahmen eines STI-Tests etwas in Ihren Anus (Analtupfer) eingeführt?

4.5 HOMOPHOBER MISSBRAUCH

Wann wurden Sie das letzte Mal angestarrt oder bedroht, weil jemand wusste oder vermutete, dass Sie sich zu Männern hingezogen fühlen?
Wann wurden Sie das letzte Mal beleidigt, weil jemand wusste oder vermutete, dass Sie sich zu Männern hingezogen fühlen?
Wann wurden Sie das letzte Mal geschlagen oder getreten, weil jemand wusste oder vermutete, dass Sie sich zu Männern hingezogen fühlen?

5.0 BESCHREIBUNGEN

5.1 DEMOGRAPHIE

Was ist Ihre derzeitige Geschlechtsidentität?
Welches Geschlecht wurde Ihnen bei der Geburt zugewiesen (Geburtsurkunde)?
Wie alt sind Sie?
In welchem Land leben Sie derzeit?
Wie lauten die ersten zwei Ziffern Ihrer Postleitzahl?
Wie groß ist der Ort, wo Sie leben?
Mit wem leben Sie zusammen? Was ist Ihr höchster Bildungsabschluss?
Welche der folgenden Aussagen beschreibt Ihren aktuellen Beruf am besten?
Wurden Sie in Ihrem aktuellen Wohnland geboren?
In welchem Land wurden Sie geboren?
Wie viele Jahre leben Sie schon in Ihrem aktuellen Wohnland?
Warum sind Sie dorthin gezogen?
Wie viele Jahre haben Sie seit Ihrem 16. Lebensjahr in Schule, Ausbildung oder (Fach)Hochschule zugebracht?
Was beschreibt Ihre derzeitige berufliche Situation am besten?
Welche der folgenden Aussagen entspricht Ihrer gefühlten Einkommenssituation am besten?
Fühlen Sie sich als Teil einer ethnischen Minderheit in Ihrem Wohnland?

5.2 SEXUALITÄTSBESCHREIBUNG

Zu wem fühlen Sie sich sexuell hingezogen?
Welche der folgenden Optionen beschreibt am besten, wie Sie sich selbst definieren? [sexuelle Identität]
Wenn Sie an die Menschen denken, die Sie kennen (Familie, Freund_innen, Arbeitskolleg_innen, Mitstudierende), welcher Anteil von ihnen weiß, dass Sie sich zu Männern hingezogen fühlen?

5.3 STATUS DER SEXUELLEN BEZIEHUNG

Leben Sie derzeit in einer festen Beziehung?
Wann hat Ihre letzte feste Beziehung geendet?
Wie lange besteht die feste Beziehung mit diesem Mann?
Ist Ihr fester Partner HIV-positiv?
Wie lange besteht die feste Beziehung mit dieser Frau?
Ist Ihre feste Partnerin HIV-positiv?
Wie lange besteht die Beziehung zu diesem geschlechtsneutralen/nichtbinären Menschen?
Ist Ihr geschlechtsneutraler/nichtbinärer Partner HIV-positiv?
